# Exploring factors hindering the uptake of HIV pre-exposure prophylaxis by potential users in Namibia

**DOI:** 10.4102/sajhivmed.v25i1.1561

**Published:** 2024-05-21

**Authors:** Daniel O. Ashipala

**Affiliations:** 1 Department of General Nursing Science, School of Nursing and Public Health, Faculty of Health Sciences and Veterinary Medicine, University of Namibia, Rundu, Namibia

**Keywords:** HIV, pre-exposure prophylaxis, uptake, barriers, Namibia, qualitative, substantial risk

## Abstract

**Background:**

Pre-exposure prophylaxis (PrEP) is recommended for people who are at substantial risk of HIV infection, in conjunction with other HIV prevention tools and strategies. Unfortunately, the uptake of PrEP among potential users in Namibia’s Okongo district is low.

**Objectives:**

The objective of this study was to explore and describe the factors that hinder the uptake of HIV PrEP by potential users at the Okongo District Hospital in Namibia.

**Method:**

A qualitative exploratory, descriptive and contextual design was used. The study sought to understand the factors hindering uptake of HIV PrEP by potential users in the peri-urban setting of Okongo District Hospital, Namibia. Purposive sampling was used to select participants for this study, with qualitative data being gathered from semi-structured interviews with 20 potential PrEP users. The data were analysed using qualitative thematic analysis.

**Results:**

Participants reported numerous factors hindering uptake, including level of understanding, non-compliance on the part of the health department, distances, and attitudes. Additionally, participants maintained that health workers do not put enough effort into educating patients in the different hospital departments about PrEP, and sometimes there is insufficient stock of the medicine, both of which hinder PrEP uptake.

**Conclusion:**

Despite the PrEP rollout, multiple obstacles continue to hinder PrEP uptake, especially in the outskirts of Okongo district. This study recognises that there is a need to work hand in hand with the support systems of both potential users.

**What this study adds:** The findings of this study provide insights into the issues that policymakers and healthcare workers should consider in order to increase and sustain PrEP uptake among potential users.

## Introduction

In 2016, the United Nations (UN) General Assembly stated that an expedited plan would have to be followed if AIDS were to cease being a threat to public health by 2030. This plan included three milestones to be reached by 2020, one of which was to reduce new HIV infections to under 500 000 globally per year. In 2022, there were an estimated 1.3 million new HIV infections among all populations.^1^ One of the strategies to make this milestone a reality was the implementation and scaling up of pre-exposure prophylaxis (PrEP) as part of an ‘HIV prevention package’.^1^ The antiretroviral (ARV) drugs that are recommended by the World Health Organization (WHO) to be used as PrEP are Tenofovir and Emtricitabine (TDF/FTC). This medicine can be used by all adults who have a high risk of contracting HIV, such as HIV-negative people whose sexual partners either have HIV or who do not know their HIV status, or those whose sexual partners may be having intercourse with others.^2^ However, before using PrEP, a patient must agree to being tested regularly for HIV to rule out infection, because if PrEP is initiated in persons who are already HIV-positive, it could result in the emergence of resistance to the drugs.^3^ Trials have yielded efficacy estimates ranging from 44% to above 90% depending on medication adherence.

The Namibia Population-Based HIV Impact Assessment (NAMPHIA) 2017 report noted that HIV prevalence among Namibian adults aged 15 years to 64 years was 12.6%, with a higher percentage observed among women and girls at 15.7% compared to 9.3% in men and boys.^4^ According to the NAMPHIA report, the annual rate of new HIV infections is estimated to be approximately 4500 among people aged 15 years to 64 years.^5^ The Ohangwena region has one of the highest HIV prevalence rates in Namibia at 17.9%, the second highest in the country.^5^ According to the NAMPHIA report, the HIV incidence is highest among the age group 15 years to 24 years.^5^

A large number of studies have been conducted on both the facilitators and barriers that affect the use of PrEP.^6^ In the context of Namibia, PrEP rollout started in 2017; however, the uptake has been slow, with only 200 users in 2018.^7^ According to the annual report of Ohangwena Regional Health Directorate, Okongo District Hospital PrEP uptake for the last 4 years was as follows: 194 new initiations in 2020, 188 in 2021, 158 in 2022, and 195 in 2023.^8,9,10,11^ The uptake for PrEP at this hospital has been low in the last 4 years considering that the catchment population is 29 999, and the number on ARV therapy (ART) gave an indication that there might be HIV-negative people who might be at risk of acquiring HIV. There is a paucity of PrEP research on the low uptake of HIV PrEP by potential users in Namibia. The low levels of PrEP uptake in Okongo district implies that there are factors that may be overlooked in the provision of PrEP. The aim of the study was to explore the factors hindering the uptake of HIV PrEP by potential users in Namibia in order to inform PrEP uptake and continuation strategies.

## Research methods and design

### Research design

A qualitative, explorative, descriptive and contextual research design was utilised in order to allow the researcher to gain more information on the phenomena under study. This design enables a researcher to assess how people understand their experiences, understandings and surroundings related to a specific phenomenon.^12^ As per Hunter et al.,^13^ the aim of explorative research is to ‘understand the underpinnings of specific phenomena and explain specific and systematic relationships among them so that they are described in rich detail’. Exploratory research intends to provide an understanding of the issue the researcher is examining. while descriptive research intends to provide details on a phenomenon and its features.^14,15^ For these reasons, this was considered to be a suitable design for assessing the factors hindering the uptake of PrEP by potential users at the Okongo District Hospital in Ohangwena Region, Namibia. A qualitative approach was used because it can aid in the investigation of themes such as life experiences.^16^ Moreover, as per Maree and Molepo, qualitative research ‘design is naturalistic, that is, it focuses on natural settings where interactions occur’.^17^

### Setting

This study was conducted at Okongo District Hospital in Ohangwena Region, Namibia. Ohangwena region is one of the 14 regions in Namibia with three districts. Okongo District Hospital is in the northern part of Namibia bordering Angola, Oshikoto region to the south, Kavango West to the east, and Oshikunde to the west. The estimated catchment population of the Okongo district is 29 999. The number of patients on ART is 3314. Data were collected from 01 November 2020 to 31 December 2020.

### Recruitment and study population

During recruitment, the researcher introduced herself to the participants, who gave their written consent to participate in the research. They were also informed of their right to withdraw from the study at any time if they so wished. The researcher made arrangements with the nurse manager and nurse in charge to identify a suitable environment to carry out the data collection. The participants were identified through their hospital records. During the interviews, the researcher used an audio recorder while also taking notes on what the participants said. Convenience sampling was used to select the participants. Participants were eligible for participation if they were: (1) patients seeking PrEP services at the Okongo District Hospital at the time of the study and (2) able to provide informed consent in English or Oshikwanyama. The sample size (*N* = 20) was chosen according to the idea of data saturation, which occurs when participants are no longer providing any substantially new or useful data, and the data categories are sufficiently developed.^18^

### Data collection

The semi-structured interviews lasted approximately 40 min to 45 min and were conducted by the researcher who has an educational background in nursing science and is fluent in English and Oshikwanyama. The researcher used an interview guide to make sure that the relevant core topics were covered, that is: (1) the participants’ understanding of PrEP, (2) their awareness of the barriers to PrEP uptake and (3) what can be done to improve the uptake of PrEP among potential users.

### Data analysis

Qualitative data are non-numerical; thus, they are typically included in the form of videos, written material, photographs and audiotapes.^19^ Basic demographic details of participants were collected. The researcher used an inductive approach to analyse the data, using the thematic analysis technique. The data gathered from the recordings were transcribed verbatim, following which they were analysed using a qualitative thematic analysis, that is, Braun’s six-step method of data analysis. This included: ‘(1) becoming familiar with the data; (2) generating initial codes; (3) coding data using nine steps; (4) determining and reporting themes; (5) defining and naming themes; and (6) interpreting the data’.^20^

### Trustworthiness

The trustworthiness of the whole study was ensured by using Lincoln and Guba’s model, which ensures the credibility, dependability, confirmability, and transferability of a study.^21,22^ Credibility was realised by holding pilot interviews, establishing data saturation, prolonging participant engagement as the researcher remained in the field for eight weeks, actively participating, observing and interacting with participants, recording the interviews, member checking (by replaying the recordings to the participants), and validating the transcripts with the research supervisor. Transferability was achieved by incorporating rich descriptions of the perspectives of the potential users, as well as their contexts, so that their meaning was evident to outsiders.^23^ Dependability was ascertained via a peer debriefing with researchers not involved in the study, as well as extended engagement with the interviewees and member checking. An inquiry audit using an external reviewer ensured confirmability. Finally, reflexivity was established by the researcher remaining aware of his roles in the study and his ongoing reflection on his personal behaviours and how these might affect the research.^22^ This reflection was made possible through the use of research diaries, which the author incorporated into his field notes.

### Ethical considerations

Ethical approval was received from the University of Namibia, Faculty of Health Sciences/School of Nursing Research and Ethics Committee (Formal Approval Number: SoNREC 37/202) and the Ministry of Health and Social Services (MoHSS) institutional review board (ref. no. JNJ 2020) before the study was conducted. Recruitment started by providing the potential participants in the district with an information explaining all the details of the study. Participation in the research was voluntary, and written informed consent was obtained from all participants.

## Results

### Participant characteristics

Table 1 shows the characteristics of the study participants. A total of 20 participants were interviewed.

**TABLE 1:** Characteristics of study participants (*N* = 20).

Characteristic	Frequency
**Age (years)**	
20	4
25	6
28	5
33	3
44	2
**Gender**	
Male	8
Female	12
**Marital status**	
Married	11
Single	9
**Employment status**	
Employed	12
Unemployed	8
**Level of education**	
Primary	2
Secondary	5
Tertiary	13

### Key themes

The three themes that resulted from the data analysis are as follows: (1) the need for prophylaxis, (2) barriers to PrEP uptake, and (3) ways to improve the uptake of PrEP. These themes and sub-themes are presented in Table 2.

**TABLE 2:** Themes and sub-themes that emerged from the data analysis.

Theme	Sub-themes
1. The need for prophylaxis	1.1 Potential users 1.2 HIV-negative protection
2. Barriers to PrEP uptake	2.1 Level of understanding 2.2 Health departments non-compliant 2.3 Distances to health facilities 2.4 Discriminated against for taking PrEP
3. Ways to improve the uptake of PrEP	3.1 Primary healthcare involvement 3.2 Advancement of the uptake 3.3 Patient commitments 3.4 Conduct training on the benefits of PrEP 3.5 Conduct outreach

PrEP, pre-exposure prophylaxis.

A detailed description of the themes and categories in this study is given below.

### Theme 1: The need for prophylaxis

This theme describes the participants’ understanding of PrEP as a prophylaxis that helps to prevent HIV infections among individuals who are at substantial risk of becoming HIV-positive, especially those having sexual intercourse with people who are known to be HIV-positive, those with an unknown HIV status such as sex workers, and men who have sex with men (MSM). The sub-themes include potential users and HIV-negative protection.

#### Sub-theme 1.1: Potential users

Participants described potential users as any HIV-negative person who is at substantial risk of acquiring HIV. This implies that the person to get PrEP should be at risk of contracting HIV and should have a negative HIV result:

‘My understanding of PrEP is that these are medicines that are taken by someone who is HIV uninfected before he/she is exposed by his/her HIV infected partner or at any time thinking they are at risk of contracting HIV. So, such persons take these medicines.’ (P7, 20 years, male)

#### Sub-theme 1.2: HIV-negative protection

In this sub-theme, participants describe the criteria or requirements that an individual should meet before they get PrEP:

‘There are criteria a person must meet to get PrEP. Like a person must be HIV negative, at risk of contracting the virus, must weigh 30 kg and above and must give consent to get tested every time they are coming for follow-up.’ (P4, 25 years, female)

### Theme 2: Barriers to pre-exposure prophylaxis uptake

This theme is a description of what study participants described as barriers to PrEP uptake among potential users. The sub-themes that emerged are level of understanding, health department’s non-compliance, distances and attitudes.

#### Sub-theme 2.1: Level of understanding

The study participants described a low level of understanding as a hindrance to PrEP access, as they felt that most potential users generally lack information about PrEP. By assessing what influences the uptake and usage of PrEP, future programmes can be adjusted to better serve the target population:

‘The other barrier is lack of information. People have no information at all about PrEP that it can protect someone from getting HIV infected if they get in sexual contacts with someone who is HIV infected.’ (P11, 28 years, female)

#### Sub-theme 2.2: Health department non-compliant

The study participants maintained that health workers do not put enough effort into educating patients in the different hospital departments about PrEP, and sometimes there is insufficient stock of the medicine, both factors that hinder PrEP uptake:

‘Hospitals have different departments and each department provide different services, some health workers might not have the information about PrEP, because they don’t work at departments were PrEP is provided.’ (P7, 20 years, male)

#### Sub-theme 2.3: Distance to health facilities

Participants revealed that some potential users of PrEP may not benefit from such services as they live far away from the health facilities where PrEP services are offered. In addition, sometimes there is no transport to take them to the hospital or they do not have the fare required:

‘Sometimes it is distance, like we know here, there are long distances from the bushes to reach the health facilities. Sometimes there are no transport at some villages, a person might be willing to get the services, but might not have transport fare or no transport at all to reach the health facilities.’ (P11, 28 years, female)

#### Sub-theme 2.4: Discriminated against for taking pre-exposure prophylaxis

Under this sub-theme, study participants described discrimination as one of the barriers that hinder PrEP uptake. This relates mainly to the beliefs that other people may have about someone who is taking PrEP or seeking PrEP services:

‘The barriers are that, like here at Okongo, these medicines are given from the ART clinic, where people are together with those already on ART. Sometimes people have fear that if they are seen at ART clinic, people will think he or she is also living with HIV and is getting ARVs to treat HIV.’ (P12, 28 years, male)

### Theme 3: Ways to improve pre-exposure prophylaxis uptake

Under this theme, participants suggested ways in which to improve the uptake of PrEP among potential users. It is vital to enhance healthcare workers’ training in this regard and to consider other service delivery models that could meet the needs of patients while improving the rate of PrEP uptake for those at risk. The following sub-themes emerged from this theme: Primary healthcare (PHC) involvement, advancement of uptake, patient commitment, conducting training and conducting outreach.

#### Sub-theme 3.1: Primary healthcare involvement

Participants mentioned that if PrEP services are integrated into other departments in the hospital, these services would be readily accessible and could improve their provision. They also noted that if everyone were educated about and made aware of PrEP services, the uptake would improve:

‘I think it will help if PrEP can also be provided at the primary healthcare (PHC) clinic where other services are provided. I think it will be better at the PHC as no one will assume what clients came for, whether they came for general treatment, family planning or they came for PrEP.’ (P16, 33 years, female)

#### Sub-theme 3.2: Advancement of uptake

Here participants suggested different strategies that can be undertaken in order to implement the effective uptake of PrEP, such as employing community PrEP mobilisers, distributing posters with PrEP information, and taking PrEP closer to the people in the community:

‘I think the Ministry needs to strengthen provision of information, by employing people to conduct mobilisation about PrEP in the communities and also to provide awareness to people who bully others about PrEP to stop doing so.’ (P2, 30 years, male)

#### Sub-theme 3.3: Patient commitment to pre-exposure prophylaxis

Under this sub-theme, participants expressed that the patients themselves have to be committed to take PrEP. They felt that for the successful implementation of PrEP, patients have to play a major role in accessing, adhering and continuing their treatment:

‘I think what will improve the service more is to make people understand, because if they understand well, they will be able to feel free to take these medicines.’ (P18, 44 years, male)

#### Sub-theme 3.4: Conduct training on the benefits of pre-exposure prophylaxis

Under this sub-theme, participants felt that healthcare workers and health extension workers (HEWs) need more knowledge about PrEP in order to provide the treatment and improve its uptake, thus they suggested that training be held to capacitate HEWs with more information:

‘I suggest that, if it is possible, health extension workers (HEWs) supposed to get PrEP training, because it’s easier in the villages as some people may be raped but can’t even come to hospital or police to report, but if the HEW is trained they will be able to help such clients.’ (P5, 28 years, female)

#### Sub-theme 3.5: Conduct outreach

Some participants believed that conducting community campaigns, giving health education, and disseminating information through the radio and television might improve PrEP uptake. One participant also suggested taking PrEP medicine to outreach services:

‘We need to give health education to alert everyone, at least they should not only be given health education when they come to seek for help but the community need to be mobilised so that they will know where to go when they are in need especially at outreach teams.’ (P10, 20 years, female)

## Discussion

This study’s findings have shown that there are barriers hindering uptake of PrEP; therefore, the results of this study project a possible ongoing HIV transmission which could inform a need for PrEP services to those considered at risk of contracting HIV in the Okongo district. To address this urgent need, Gombe et al. highlight that it may be necessary to consider perspectives and preferences of PrEP users as this can help meet their needs.^24^ Gombe et al.^24^ further state that ‘the effectiveness of PrEP is dependent on its acceptability, accessibility, adoption and sustainability as part of a comprehensive HIV prevention package’. To address the barrier related to accessibility, WHO in 2015 recommended replacing its previous recommendations to enable PrEP to be offered to all those at high risk of contracting HIV, rather than just a few groups.^7,25^ In addition, WHO further encouraged clinics to offer PrEP based on individual assessments, rather than risk groups in order to foster implementation.^25^

This study further lists additional barriers hindering the uptake of PrEP, including level of understanding, a lack of compliance by the health department, distances and attitudes. Dang et al. propose a training for healthcare providers in order to strengthen their capacity on how to render an inclusive and affirming care which is considered one of the key areas for intervention to enhance the use of PrEP.^26^ Due to the geographic location, some people live in remote areas with limited to no transport or road infrastructure, which limits their access to PrEP services. On the other hand, Taggart et al. link the lack of knowledge about barriers towards the uptake and ongoing usage of PrEP in the British HIV-positive communities to issues like inequalities in HIV literacy in areas affected by HIV.^27^ This could justify why the majority of potential users are still unaware of these services, and why only a few people seek to access the service. Mayer et al. affirm that distance to clinics is also an impediment to PrEP uptake and ongoing usage in a rural community in Uganda, that is, there is a trend towards a lower likelihood of clinic attendance among people who have a longer walking distance and difficulty accessing transport.^28^

Some participants in this study also felt dissatisfied with inadequate supply of PrEP mediations which was a discouragement to some patients in returning for the next follow-up. In events of adequate supply of PrEP medications, most healthcare facilities may yet be confronted with a challenge of limited finance and human resources as barriers to PrEP implementation in general.^29^ Moreover, Jackson-Gibson et al. report the side effects of PrEP medications as a barrier to the implementation, medication uptake and persistence.^29^ Some participants reported that since these medicines are collected from the ART clinic together with those on lifelong HIV treatment, some potential users may not seek these services in fear of being viewed as HIV-positive. In the United Kingdom, major barriers identified were low awareness of PrEP and HIV-related stigmatisation; therefore, participants preferred a trusted community setting that is suitable for HIV testing and prevention discussions.^29,30,31^

It emerged from this study that several interventions need to be undertaken to improve the uptake of PrEP within the Okongo district. For example, strategies like intense PHC involvement can help advance the uptake of PrEP services given that PHC facilities are considered the first point of healthcare entry. Gombe et al. suggest integrating PrEP services into family planning clinics for easy accessibility.^24^ Marcus et al.^32^ also suggest that healthcare facilities should have well-developed tools and techniques that clearly provide information about PrEP to potential patients, while providing support to health providers on how to use PrEP.^32^ In addition, there is a need to maximise persistence with PrEP by developing approaches for younger PrEP users, minimise logistical barriers and devise effective communication on side-effect management.^33^

Participants in this study mentioned that one of the ways to improve PrEP uptake is to improve the commitment of participants to PrEP, by raising awareness on the benefits of PrEP and decentralising PrEP services. Participants further suggested the use of posters or pamphlets with clear messages about PrEP, preferably in local languages so that potential users can clearly get the message. This study’s findings demonstrated the importance of healthcare workers counselling their patients about sharing the importance of PrEP with their loved ones for sustained effective use.^34^ Similarly, a study done in Kenya also noted the critical roles played by healthcare workers in counselling patients on PrEP use, including alternative means of prevention and community awareness.^35^ However, the need to consistently build capacity of healthcare workers on PrEP is key to enable them to dispatch correct PrEP information to PrEP users through awareness campaigns in the community, radio and TV channels.

### Strengths and limitations

A sufficient number of participants were interviewed to reach data saturation. A limitation is that although a qualitative finding provides detailed, contextual information, this study’s findings may not be generalisable outside of the population of participants interviewed in Okongo, Namibia. The use of convenience sampling for people seeking PrEP might have introduced bias, as participants may not be fully representative of all people in the district. Additionally, the study’s reliance on self-reporting through interviews may lead to social desirability bias, where participants provide responses that they perceive to be socially acceptable. Triangulation of data from multiple sources or methods could enhance the credibility and validity of the findings. The study could benefit from incorporating perspectives from nurses, doctors and other stakeholders involved in PrEP provision. The study is limited by a lack of available published data on the status of uptake of PrEP at the Okongo District Hospital. Data were only available in the hospital-based reports and regional annual reports which are also not available online.

## Conclusion

Findings from this study revealed that the rollout of PrEP is facing multiple barriers that continue to hinder PrEP uptake, especially in the Okongo district. These barriers include level of understanding, non-compliance on the part of the health department, distances and attitudes. Findings from this study recognise that there is a need to work hand in hand with the support systems of both potential users and providers to deal with these barriers so as to increase the uptake of PrEP. The findings of this study provided crucial insights into the issues that policymakers, the Ministry of Health and Social Services and healthcare workers should consider in order to increase and sustain PrEP uptake among potential users. Data from this study could be used by the Ministry of Health and Social Services to institute ongoing strategies and targeted interventions that may help potential users and providers to better utilise this medication.
